# Endometrial cancer and associated risk factors among non-pregnant women with abnormal uterine bleeding in Kampala, Uganda: a 7-year experience from a Single Institution

**DOI:** 10.4314/ahs.v25i2.18

**Published:** 2025-06

**Authors:** Betty A Kassimo, James J Yahaya, Emmanuel Othieno, Livex A Okwi, Michael Odida

**Affiliations:** 1 Department of Pathology, Faculty of Health Science, Busitema University, Mbale, Uganda; 2 Department of Pathology, School of Health Sciences, Soroti University, Soroti, Uganda; 3 Department of Pathology, Makerere College of Health Science, Kampala, Uganda; 4 Department of Pathology, Faculty of Medicine, Gulu University, Gulu, Uganda

**Keywords:** Abnormal uterine bleeding, endometrial cancer

## Abstract

**Introduction:**

Endometrial cancer is the most common malignancy of among postmenopausal women in high income countries, however, its prevalence has been reported to be raising even in low-and middle-income countries due to change in lifestyle behaviors. We aimed to determine the prevalence of endometrial cancer and its associated factors among non-pregnant women with abnormal uterine bleeding.

**Methods:**

After obtaining ethical approval from the Research Ethical Committee of the Makerere college of Health Sciences, we retrospectively retrieved and analyzed the paraffin embedded tissue blocks of 159 patients who were non-pregnant women and they were presenting with abnormal uterine bleeding from January 2012 to December 2018. The clinical and pathological information was obtained from the patient's files. Statistical analysis was done using SPSS version 23.0. Binary logistic regression analysis was applied in determining the risk factors of endometrial cancer. A two-tailed p -value <0.05 was considered significant.

**Results:**

The overall mean age of the patients was 36.4 ± 13.7 years and the vast majority 71.1% (113/159) of the patients were premenopausal women. The prevalence of endometrial cancer was 11.9% (19/159). Being postmenopausal (AOR = 7.7, 95% CI = 2.155 – 27.666, p = 0.003), having menarche below 12 years (AOR = 4.5, 95% CI = 0.217-0.390, p<0.001), being obese (AOR = 2.5, 95% CI = 0.305-0.822, p = 0.01), and being nulliparous (AOR = 5.4, 95% CI = 0.292-0.957, p = 0.009) were significantly associated with endometrial cancer. However, use of contraceptive pills, family history of reproductive cancers, having hypertension and/or diabetes mellitus all were not associated with endometrial cancer.

**Conclusion:**

Our study has further proven that, there is a significant proportion of non-pregnant women with AUB who have endometrial cancer. Using the clinical parameters such as age of the patient and age at menarche, nulliparity and obesity, it may help in increasing the index of clinical suspicion in daily clinical practice to diagnosing endometrial cancer among women with AUB at early stage. This will help in improving the prognosis of the patients.

## Introduction

Endometrial cancer (EC) is the fifth leading cancer among women worldwide with 320,000 new cases diagnosed in 2012, which accounts for 4.8% of all cancers in women^1^. Globally, approximately 10% of all premenopausal and postmenopausal women with abnormal uterine bleeding (AUB) are diagnosed with endometrial carcinoma (EC) which contributes to significant maternal morbidity and mortality^2^. According to Gon et al., in 2013 it was reported that, there is increasing risk of EC among women who present clinically with AUB and the vast majority of them undergo hysterectomy without a definite diagnosis^3^.

EC is the most common gynecological malignancy in high income countries (HICs) unlike low-and middle-income countries (LMICs) in which malignancy of uterine cervix is the leading gynecological malignancy^3^,^4^ and its incidence has been observed to be increasing exponentially for the past 10 years^5^. In the pas, EC was known to be rare among premenopausal women, however, with increasing obesity and raising prevalence of metabolic syndrome, the incidence of EC has been found to increase even in low-and middle-income countries (LMICs)^3^. Another possible reason attributed to the observed high prevalence of EC in HICs could be availability of sufficient and reliable pathology services^6^. Lack of pathology services in most low-and middle- incomes countries like Uganda, causes the incidence of EC in such countries to be underrated^6^. In a study which was done in Cameroon among women with AUB, the prevalence of EC was reported to be 7.8%^6^. Another study by Tolu et al reported prevalence of EC of 4.7% among women with AUB in Ethiopia^7^. In another study which was done in Nigeria, the prevalence of EC among women with AUB was found to be 10.1%^8^. Also, Asuzu and Olaofe reported a less than 1 percent prevalence of EC from histopathological biopsies among women with AUB in Nigeria (Asuzu & Olaofe 2018).

The main risk factor for EC is an excess of endogenous or exogenous estrogen without adequate opposition by progesterone^10^. Studies suggest that having many pregnancies may protect against EC compared to women who have never given birth (Hale et al. 2002; Mahmoud & Rifat 2013; Whitaker & Critchley 2016). During pregnancy, the hormonal balance shifts toward more progesterone^12^. Elevated progesterone may inhibit estrogen associated endometrial cell proliferation and promote endometrial cell differentiation and apoptosis^13^,^14^. In a study which was done by Bakkum-Gamez et al it was found that, unopposed exposure to estrogen is associated with increased risk of EC development^15^. Chronic anovulation, polycystic disease (PCOD), generalized obesity, nulliparity and type 2 diabetes mellitus (T2DM) have been found to be positively associated withncreased risk of EC (Hale et al. 2002; Jayawickcrama & Abeysena 2019; Nagy et al. 2009). Despite the number of women with AUB observed in Uganda, currently, there is no data which address the prevalence of EC in such a population. Additionally, although the number of risk factors that have been cross-examined with EC in the present study have been studied in many settings globally, such risk factors have never been associated with EC in Uganda particularly in a cohort of non-pregnant women with EC. By investigating on this important particular subject, it will help in bringing upfront a number of risk factors which may be used to improve the index of clinical suspicion of EC that present with AUB clinically. Therefore, the primary objective of our study was to investigate the prevalence of EC and the secondary objective was to assess the risk factors associated with EC among women with AUB.

## Methods

### Study design and setting

This was a cross-sectional analytical clinical-based study. The study involved quantitative approach by retrospectively reviewing recorded clinical and retrieving and re-analyzing paraffin embedded tissue blocks of non-pregnant women with AUB. The study was conducted at the department of pathology, Makerere College of Health Science (MakCHS) which is the constituent of the Makerere University.

### Patients' characteristics and recruitment

We included clinical and pathological retrospective data of 159 patients. The patients were non-pregnant women who were presenting clinically with AUB and in whom endometrial curretings were sampled and submitted for histopathological evaluation from January 2012 to December 2018. The inclusion criteria included being non-pregnant and having complete clinical data, and available paraffin embedded tissue blocks for re-evaluation. On the other hand, all patients with incomplete clinical information, missing paraffin embedded tissue blocks, and those that had a record of being pregnant were all excluded.

### Sampling procedure and data collection

Convenience sampling method was used in selecting the cases that were included in the present study. All patients that met the inclusion criteria were included in the study. All the extracted clinical and pathological data from the patients' files were used to study the patients' files were included in the analysis.

### Data analysis

Data were analyzed using SPSS version 23.0 (IBM Statistics, Chicago, USA). Continuous variables were presented in mean ± standard deviation (SD) whereas categorical variables were summarized in frequency and percentage. Descriptive statistics were used to present the frequencies and proportions. Inferential statistics were used to deduce the association of differet variables. We determined the risk factors of EC using conditional binary logistic regression in which only variables that were significant and those with p<0.2 under univariate analysis were fitted into multivariate analysis for controlling of confounders. The confidence interval (CI) was calculated at 95% with odds ratios (OR). A two-tailed p< 0.05 was considered statistically significant.

## Results

### Selection process of the patients included in the present study

**Figure 1:**
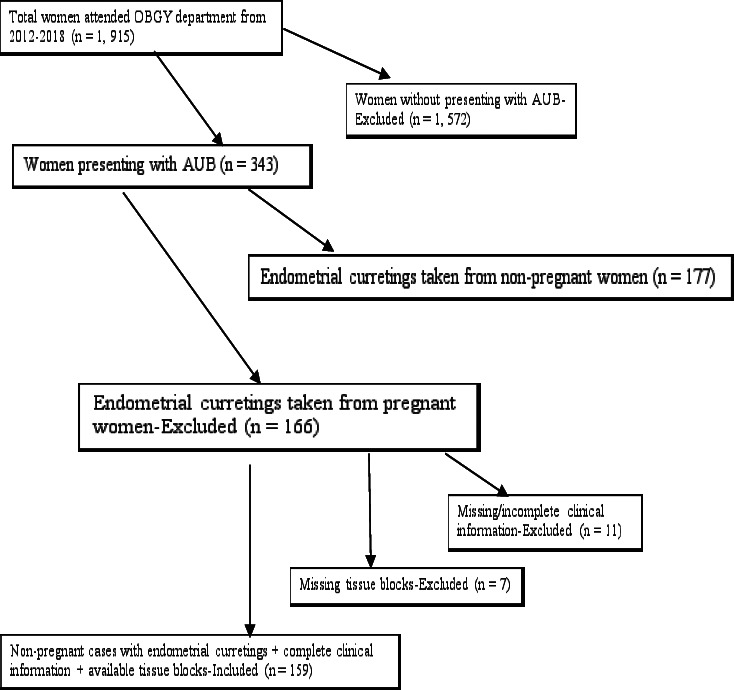
Chart flow indicating selection process of the patients included in the study

Figure 1 shows the chart flow of the selection process of the patients who were analyzed. From 2012 to 2018, a total of 177 non-pregnant women with AUB underwent endometrial curretings. Of all women who underwent endometrial currentings, 10.2% (18/177) were excluded from the study, making the retaining rate for the study to be 89.8% (159/177).

### Background information of the patients

This study reports on a cohort of 159 non-pregnant women with AUB. The overall mean age of the patients was 36.4 ± 13.7 years (range: 15-80 years). On the other hand, the mean age for premenopausal and postmenopausal was 29.7 ± 8.1 years (range: 15-50 years) and 54.3 ± 3.3 years (51-80 years), respectively. Majority of the patients 56% (89/159) were premenopausal and 41.4% (50/159) of the women were postmenopausal. Also, most of the patients 35.8% (57/159) were in age group of 25 – 34 years. Over one-quarter of the patients 25.8% (41/159) were obese and also 20.8% (33/159) were nulliparous. Concerning education for the patients, most of the patients 37.7% (60/159) had attained primary school education. Description of other variables is as detailed in Table 1.

**Table 1:** Background information of the patients recruited for the study (N = 159)

Variables	Frequency (n)	Percentage (%)
**Age (years)**		
Premenopausal age (≤42)	89	56.0
Perimenopausal age (43-50)	20	12.6
Postmenopausal age (>50)	50	41.4
**BMI (kg/m^2^)**		
Underweight (<18.5)	8	5.0
Normal (18.5-24.9)	90	56.6
Overweight (25-29.9)	20	12.6
Obese (≥30)	41	25.8
**Marital status**		
Single	38	23.9
Married/cohabiting	79	49.7
Separated/divorced	27	17.0
Widows	15	9.4
**Place of residence**		
Rural	90	56.6
Urban	69	43.4
**Level of education**		
Informal	11	6.9
Primary	60	37.7
Secondary	53	33.3
Tertiary	35	22.0
**Occupation**		
Unemployed	49	30.8
Self-employed	71	44.7
Employed	39	24.5
**Parity**		
Nulliparous	33	20.8
Primipara	23	14.5
Multipara	56	35.2
Grandpara	47	29.5
**Comorbidities**		
Hypertension	21	13.2
Diabetes mellitus	18	11.3
Both hypertension and diabetes mellitus	4	2.5
Peptic ulcer disease	16	10.1
Never	82	51.6
Unknown	18	11.3

### Clinical characteristics of the patients included in the study

**Figure 2:**
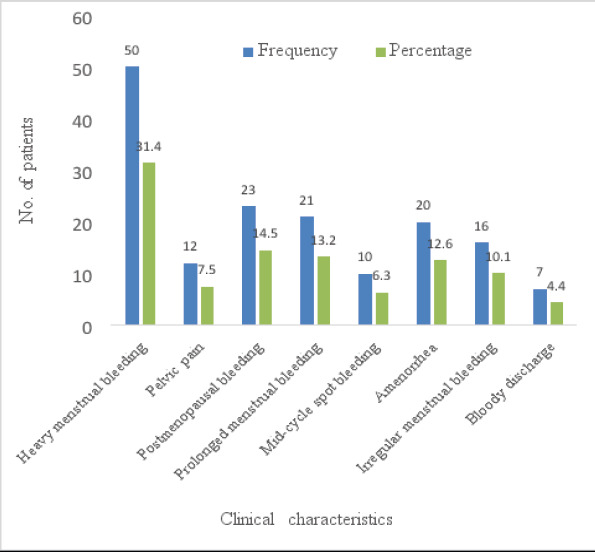
Clinical characteristics of the patients analyzed in the study

Heavy menstrual bleeding was the most common clinical characteristic which comprised 31.4% (50/159) of the all the patients followed by postmenopausal bleeding which constituted 14.5% (23/159). The least clinical characteristic was per vaginal bloody discharge which was found in only 4.4% (7/159). Other clinical features are detailed in Figure 2.

### Prevalence of endometrial cancer among patients analyzed in the study

Table 2 presents the histopathological findings of the endometrial currentings of patients included in the analysis. The prevalence of endometrial cancer was 11.9% (19/159). Simple endometrial hyperplasia was the most common histopathological diagnosis which consisted of 19.5% (31/159) followed by complex endometrial hyperplasia without atypia which was found in 16.9% (27/159) of all the included cases.

**Table 2:** Histopathological findings among patients presenting clinically with AUB (N = 159)

Histopathological findings	Frequency (n)	Percentage (%)
Simple endometrial hyperplasia	31	19.5
Complex endometrial hyperplasia without atypia	27	16.9
Complex endometrial hyperplasia with atypia	15	9.4
Endometrial cancer	19	11.9
Endometrial atrophy	12	7.5
Chronic endometritis	9	5.7
Arias-Stella reaction	4	2.5
Proliferative phase of endometrium	15	9.4
Secretory phase of endometrium	9	5.7
Endometrial polyps	15	9.4

### Risk factors of endometrial cancer among patients included in the study

There was a positive association between age of the patients with EC. Patients who were postmenopausal were almost 8 times more likely to develop EC compared with patients who were premenopausal and the difference was significant (AOR = 7.7, 95% CI = 2.155-27.666, P = 0.003). Non-pregnant women who got menarche while younger than 12 years were almost 5 times moe likely to develop EC than women who got menarche at ≥12 years and the difference was significant (AOR = 4.5, 95% CI = 0.217-0.390, p<.001). Increased BMI to a level of being obese had a 2.3-fold increased risk of contributing to the development of EC among patients with statistical difference (AOR = 2.3, 95% CI = 0.305-0.822, p = 0.01). Being nulliparous had a 5.4-fold increased risk of EC compared with being parous and the difference was significant (AOR = 5.4, 95% CI = 0.292-0.951, p = 0.009). Also, we found that being nulligravida had a 3.4-fold increased risk of developing EC compared with being grandgravida (having ≥5 pregnancies) although the difference was not significant (AOR = 3.4, 95% CI = 2.331-11.023, p = 0.08). Interestingly, we found that women who were primigravida (AOR = 0.5, 95% CI = 0.712-4.334), p = 0.207) or multigravida (AOR = 0.8, 95% CI = 0.712-1.951), p = 0.114), both were protected against developing EC but not significantly (Table 3).

**Table 3:** Binary logistic regression analysis of risk factors of endometrial cancer

	Univariate analysis	Multivariate analysis
**Variables**	**COR (95% CI), p-value**	**AOR (95% CI), p-value**
**Age (years)**		
Premenopausal age (≤42)	1 [Reference]	1 [Reference]
Perimenopausal age (43-50)	1.7 (0.451-2.932), 0.301	1.1 (0.382-10.604), 0.911
Postmenopausal age (>50)	3.2 (0.070-0.845), 0.02	7.7 (2.155-27.666), 0.003
**Age at menarche (years)**		
<12	1.8 (1.108-5.792), 0.004	4.5 (0.217-0.390), <0.001
≥12	1 [Reference]	1 [Reference]
**BMI (kg/m^2^)**		
Not obese (<35)	1 [Reference]	1 [Reference]
Obese (>35)	4.0 (0.963-3.772), 0.109	2.3 (0.305-0.822), 0.01
**Use of contraceptive pills**		
Never	1 [Reference]	1 [Reference]
Ever used/using	1.0 (0.258-6.004), 0.407	-
**Nulliparous**		
Yes	2.7 (1.311-12.402), 0.002	5.4 (0.292-0.951), 0.009
No	1 [Reference]	1 [Reference]
**Gravidity**		
Nulligravida	1.8 (0.408-0.811), 0.01	3.4 (2.331 -11.023), 0.08
Primigravida	0.8 (0.112-3.709), 0.08	0.5 (0.712-4.334), 0.207
Multigravida	0.3 (0.206-7.332), 0.07	0.8 (0.712-1.951), 0.114
Grandgravida	1 [Reference]	1 [Reference]
**Diabetes mellitus**		
Yes	0.9 (0.622-9.118), 0.703	2.3 (0.505-3.061), 0.07
No	1 [Reference]	1 [Reference]
**Hypertension**		
Yes	1.3 (1.040-17.208), 0.012	2.5 (0.661 -5.704), 0.331
No	1 [Reference]	1 [Reference]
**Family history of reproductive cancers**		
Yes	1.6 (0.409-10.115), 0.305	-
No	1 [Reference]	1 [Reference]

## Discussion

Studies reporting on the magnitude of EC in LMICs are generally scarce. This scarcity has contributed to underreporting of EC in most of LMICs including Uganda. We present findings on prevalence and associated risk factors of EC from a cohort of women who were presenting clinically with AUB.

The prevalence of EC of 11.9% which was observed in the present study is quite higher than a range of 4.8%-10.1% of EC that was reported in previous studies^6^,^7^,^11^,^17^. Also, lower prevalence of EC ranging from 0.2%-4.4% has been reported elsewhere 18–20. Furthermore, lower incidences of 3.9% and 3.14% of EC among women with AUB has been reported in previous studies^21^,^22^. However, higher prevalence of EC of 21% and 17.6% than the one observed in our study^23^,^24^. The lowest incidence of EC reported in other studies may be due to inclusion of many patients with younger age but also the variation in percentage of the known risk factors for EC like parity and generalized obesity among study participants across studies reported previously. Availability of predisposing factors such as endometrial hyperplasia with or without atypia as well as polycystic ovarian diseases (PCODs) may help to explain the variation in the prevalence of EC.

In the present study there was a strong positive association between age and EC among study participants. We observed that women who had 50 years and above (postmenopausal) were almost 8 times more likely to develop EC compared with those whoad less than 50 years (premenopausal). This observation has also been reported in other studies done elsewhere. In studies of Afghan et al. and Abdullah et al. both reported higher risk of EC among women with AUB who were aged more than 40 years compared with those who had less than 40 years^25^,^26^. Chen et al. also observed that women with age more than 50 years were almost 3 times more likely to develop EC than the ones with less or equal to 50 years^27^. Old age (more than 50 years) which is a postmenopausal age is associated with increased risk of many malignancies among women not only EC^28^. This fact also explains why EC is more common in HICs due to long life expectancy and indulging in more other risk behaviors such as smoking and being obese among many others^29^. Because of short life expectancy in LMICs, this may be the reason for the observed low prevalence of EC^30^. Another possible reason for advanced age being a non-modifiable high risk of EC is due to accumulation of mutations, epigenetic alterations and other genomic instabilities which all together account for the increased risk of EC^31^.

Furthermore, we found that, women who had had attained menarche while younger than 12 years, had almost a 5-fold significantly increased risk of developing EC compared with their counterparts. Studies have shown that both early age at menarche (less than 12 years) and late post-menopause (beyond 55 years) both are associated with increased risk of not only EC but also breast cancer and ovarian cancer^32^–^34^. Prolonged exposure to estrogen as a result of early menarche and late post-menopause due to lack of opposing effect of progesterone has been coined to increased risk of EC. Increased level of estrogen in the circulation acts as a nourishment for increasing proliferation of the endometrial lining as well as increasing mitotic activity of the endometrial cells^35^, this renders them susceptible to dysplastic changes and even malignant transformation^11^.

In our study, obesity was significantly associated with EC which is similar to the finding in the study of Jocelyn et al. in which it was observed that the risk of developing EC among women with BMI greater than 35.0 kg/m^2^ was 5.1 compared to 3.5 for women who had BMI less than 35.0 kg/m^2^ 36. Also, another similar finding was reported in the study of Mahmoud et al. in which only 0.2% of non-obese women had EC compared to 22.4% of obese women who had EC (p = 0.0005)^11^. Abnormal increased body weight which may be measured commonly by BMI but also waist circumference (cm) and waist to hip ratio^11^ is associated with increased deposition of adipose in the body. Obesity has been linked with excess estrogen in the circulation 34 which causes stimulation growth of the endometrial wall as well as increased mitotic activity of the lining epithelial cells^11^. Decreased levels of circulating sex binding-globulin and increased conversion of androstenedione to estrogen have been suggested the possible mechanisms through which obesity leads to increased level of free circulating estrogen in the body^36^,^37^.

We also found a positive association between being nulliparous and having EC in our study. This observation has also been frequently reported in other studies elsewhere. For example, Rifat et al reported a positive association between being nulliparous and EC in which 26.3% of nulliparous women with AUB had EC compared with 2.1% women who were parous (p = 0.006)^11^. Similarly, Al-Neaimy et al observed that, 23.8% of nulliparous women had EC compared to 9.1% of parous women who had EC (p = 0.001)^21^. Furthermore, a correlation was found on EC and nulliparity in a study which showed that, out of the 3 cases of EC that were observed, two were nulliparous of EC (P = 0.0000167)^38^. All these findings and many others indicate that, nulliparity is an important risk factor of EC. It has been postulated that, nulliparous is associated with decreased level of progesterone and increased level of free estrogen which in turn leads to increased endometrial proliferation^1^. Also, it was found that nulliparity is associated with chronic anovulation which in turn causes increased unopposed estrogen level in the circulation^11^.

Gravidity which is a number of pregnancies a woman would have in her life time was also a predictor of EC in our study. We observed that, being nulligravida was significantly associated with increased risk of developing EC. In a study was done by Manga et al. and Maryam et al. both reported significant association between nulligravida and possibility of developing EC^6^,^39^. However, in 2021 Zhao et al. reported that there was no association of nulligravidity and EC^40^. Gravidity as it is for parity both biological processes involve ovulation in which there is opposing effect of progesterone against estrogen which helps to prevent the endometrium from proliferating^11^. This helps to reduce the chance for EC from developing. Regarding use of oral contraceptive pills (OCPs) with possibility of developing EC in our study, we observed that the two variables were not associated. This is similar to the finding of the study by Manga et al.^6^. However, other previous studies have reported a positive association between use of OCPs and EC. For example, Weiss et al. reported that the odds for developing EC among study participants were increasing proportionally with the increase of both duration of using OCPs and aggressiveness of EC^36^. Furthermore, in a study done by Maryam et al. it was also observed that, there were many patients with EC who were using OCPs than those who were not using OCPs^39^. It has been argued that, duration of using OCPs and whether such OCPs contain progestin or not are key determinant in developing ECa among women^41^. This observation may apply for lack of association of use of OCPs and EC in our study because it was not possible to determine both duration of use pf the OCPs as well as whether the pills were containing progestin due to retrospective nature of the data analyzed.

Although studies have shown a positive association between family history of reproductive cancers and EC^1^,^39^, lack of association for the two variables seen in our series has also reported in another study previously^42^. For cross-sectional studies it is not possible to establish the causal-effect relation as it was in our series, this may explain for lack of association of family history of reproductive cancers and EC among participants in the present study.

As it is a rule of thumb that there is no single study without limitations, our study also had some limitations among which it was the fact that we used retrospective data which for some data were incomplete including inability of establishing the causal-effect relation. Our findings also lack generalizability because of selection bias caused by convenience sampling.

In conclusion, despite the relatively low prevalence of EC observed in our study from a lot of women with AUB, still the findings are crucial in substantiating the need of making close evaluation for women including sampling of endometrial curretings aiming at diagnosing EC when is still at early stage. This may be emphasized more for postmenopausal women particularly who present clinically with AUB due to high risk of developing EC. Women with a history of having early menarche, and those with obesity, nulliparous, and family history of reproductive cancers need to be under frequent surveillance for EC due to being at a high risk as it was shown in the present study.
